# Functional characterization of the bovine sour taste receptor Otopetrin 1 (OTOP1) in response to dietary and ruminal acids

**DOI:** 10.1016/j.bbrep.2026.102463

**Published:** 2026-01-29

**Authors:** Fuminori Kawabata, Yuko Kawabata

**Affiliations:** a Physiology of Domestic Animals, Faculty of Agriculture and Life Science, Hirosaki University, 3 Bunkyo-cho, Hirosaki, Aomori, 036-8561, Japan; b United Graduate School of Agricultural Sciences, Iwate University, 3-18-8 Ueda, Morioka, Iwate, 020-8550, Japan; c Section of Oral Neuroscience, Graduate School of Dental Science, Kyushu University, 3-1-1 Maidashi, Higashi-ku, Fukuoka, 812-8582, Japan

**Keywords:** Bovine, Cow, Sour taste

## Abstract

Cattle frequently consume acidic feeds such as silage and are continuously exposed to acidic substances originating from ruminal fermentation during rumination. These acids are presumed to be detected *via* sour taste receptors expressed in taste buds; however, the functional properties of bovine sour taste receptors remain poorly understood. In this study, we focused on the bovine sour taste receptor candidate Otopetrin 1 (OTOP1) and characterized its functional responses to physiologically relevant dietary and ruminal acids, as well as its modulation by zinc ions. Bovine OTOP1 was heterologously expressed in HEK293T cells, and its acid responsiveness was evaluated using whole-cell patch clamp and membrane potential assays. Cells expressing bovine OTOP1 exhibited pH-dependent inward currents in response to HCl. In addition, bovine OTOP1 was activated by a range of organic acids present in cattle feed and the rumen, including formic, citric, malic, tartaric, lactic, acetic, butyric, and propionic acids. Furthermore, acid-induced OTOP1 currents were inhibited by zinc ions in a concentration-dependent manner. These findings demonstrate that bovine OTOP1 can function as a sour taste receptor responsive to multiple physiologically relevant acidic compounds.

## Highlights

• Bovine OTOP1 is activated by multiple dietary and ruminal organic acids.
• Bovine OTOP1 shows distinct sensitivities to different classes of acidic compounds.
• Zinc inhibits bovine OTOP1 only at pharmacological concentrations.

## 1 Introduction

The sense of taste guides animals in choosing nutritious foods and avoiding spoiled and toxic substances, and it is closely related to their feeding behavior. Thus, elucidating taste-sensing systems in cattle can enhance our understanding of bovine nutrition and improve feeding strategies in animal husbandry. In humans and mice, it is widely accepted that tastes can be classified into five basic categories: sweet, umami, bitter, sour, and salty. Sweet taste detects carbohydrates as a source of energy, umami taste detects amino acids as a source of protein, salty taste detects minerals, sour taste detects food spoilage, and bitter taste detects toxic substances.

On the other hand, although cattle are economically important animals, there is significantly less research on bovine taste compared to humans and model animals such as mice and rats. In their review summarizing ruminant tastes, Ginane et al. noted that cattle show a particular preference for umami taste, a slight preference for sweet taste, and a varying preference for salty taste depending on mineral needs [1]. Cattle have a smaller repertoire of bitter taste receptors (19 genes) than mice (35 genes) or humans (25 genes) [2], and they are thought to be more tolerant of bitter tastes [1]. This has been proposed as a reason that herbivores, if sensitive to bitterness, would be severely restricted in their dietary options, and why they do not need to reject bitter substances as frequently, owing to the high detoxification capacity of rumen microorganisms [1]. Moreover, it has been reported that fungiform and circumvallate papillae in cattle contain many taste buds [3], and that some taste cells in these bovine papillae express α-gustducin [4,5], a taste-sense-specific G-protein subunit that particularly involves the transduction of sweet, umami, and bitter tastes in mice [6,7]. These results suggest that cattle tongues possess a sufficient number of functional taste cells.

In this study, we focused on sour taste, which cattle tend to avoid [1]. In addition, high concentrations of acetic acid, which is abundant in silage, significantly reduce silage intake in cattle [8]. The bovine chorda tympani and glossopharyngeal nerves (both of which transmit taste information to the central nervous system) show clear action potentials when an acetic acid solution is applied to the tongue [9]. These results indicate that cattle can taste sourness. A neural response analysis of bovine single chorda tympani nerve fibers to various taste solutions revealed four major clusters: nerves that best respond to salty taste, those that best respond to sour taste, those that best respond to short-chain fatty acids such as propionic and butyric acids, and those that best respond to certain bitter substances, such as denatonium; there are also nerves that respond to all five basic tastes, as in mice [10]. Ascorbic acid, found in grass, and short-chain fatty acids, which return to the oral cavity from the rumen, likely contribute to the strong neural responses observed for these acidic compounds.

Otopetrin 1 (OTOP1) is a proton-selective ion channel inhibited by zinc ions [11]. OTOP1 is expressed in PKD2L1-positive taste cells, which are classified as type III taste cells and sour cells [11]. *OTOP1* knockout mice showed a markedly attenuated gustatory nerve response to sour stimuli, indicating that OTOP1 is a functional sour taste receptor in mice [12]. On the other hand, the bovine genome also includes *OTOP1*, but we could not find any reports on the detailed function of bovine OTOP1. Bovine OTOP1 (Accession No. NP_001193713.1) is 78.08 % homologous to mouse OTOP1 (Accession No. NP_766297.2). Because these amino acid sequence differences may lead to variations in ligand sensitivity, the results obtained from mice should not be directly extrapolated to understand the sour taste perception mechanism in cattle. Thus, this study focused on the function of the sour taste receptor in cattle, a unique animal in which acidic substances enter the oral cavity both directly from the mouth and repeatedly through rumination from the rumen. Furthermore, cattle possess a regulatory mechanism that maintains the ruminal pH within the range of approximately 5.5–6.5 by secreting large amounts of saliva containing bicarbonate and phosphate buffers [13]. Therefore, it is conceivable that sour taste receptors in the oral cavity might detect decreases in ruminal fluid pH during rumination and promote salivary secretion. Thus, elucidating the function of bovine sour taste receptors is essential for understanding physiological phenomena that are unique to cattle. In particular, we aimed to elucidate the response of the sour taste receptor OTOP1 to acidic substances in feed, such as silage, and to typical acidic substances that enter the oral cavity through rumination. We found that bovine OTOP1 is activated by a variety of acids that would actually enter the oral cavity. In addition, its activity was partially inhibited by zinc ions. This information is important for understanding cattle taste physiology.

## 2 Materials and methods

### 2.1 Chemicals

Hydrochloric acid (HCl) and lactic acid were purchased from Nacalai Tesque (Kyoto, Japan). Malic acid was purchased from Tokyo Chemical Industry Co. (Tokyo). Acetic acid was purchased from Kanto Chemical Co., Inc. (Tokyo). The other chemicals were purchased from FUJIFILM Wako Pure Chemical (Osaka, Japan).

### 2.2 Construction of bovine OTOP1

The coding sequence of bovine *OTOP1* (Accession No. NM_001206784.2) was synthesized and assembled from synthetic oligonucleotides and/or PCR products (Thermo Fisher Scientific, Waltham, MA, USA). The fragment was inserted into pcDNA3.1(+). The plasmid DNA was purified from transformed bacteria, and its concentration was determined by UV spectroscopy. The final construct was verified by sequencing. The sequence identity within the insertion sites was 100 %.

### 2.3 Measurement of membrane potential change**s**

Human embryonic kidney 293 T (HEK293T) cells were maintained in Dulbecco's Eagle Medium (DMEM high glucose, 043–30085, Fujifilm Wako Pure Chemical Corporation) containing 10 % fetal bovine serum (GE Healthcare, Buckinghamshire, UK) at 37 °C under 5 % CO_2_. To express bovine OTOP1 channel, bovine OTOP1/pcDNA3.1(+) was transfected into HEK293T cells using ScreenFect A (2999–73203, Fujifilm Wako Pure Chemical Corporation). Transfected cells were seeded at approximately 21,000 cells/well into 96-well optical/black-bottom microplates (165305, Thermo Fisher Scientific) coated with poly-d-lysine (168–19041, Fujifilm Wako Pure Chemical Corporation) and cultured in DMEM containing 10 % fetal bovine serum at 70 μL/well for 48 h at 37 °C under 5 % CO_2_. The transfected cells were then incubated with an equal volume (70 μL) of membrane potential-sensitive dye solution (FLIPR membrane potential assay kit; R8042, Molecular Devices, San Jose, CA, USA) for 30 min at 37 °C. Changes in fluorescence intensity (excitation, 530 nm; emission, 565 nm) were monitored at 1.5-s intervals at 37 °C using a FlexStation 3 microplate reader (Molecular Devices). After acquiring a baseline recording for about 16 s, 35 μL 1 × HBSS buffer (Gibco, Thermo Fisher Scientific) supplemented with 5 × test solutions (1.5–50 mM HCl, 0.5–15 mM citric acid, 1.5–25 mM butyric acid, 1.5–25 mM acetic acid, 1.5–25 mM formic acid, 1.5–25 mM malic acid, 1.5–50 mM lactic acid, 1.5–25 mM propionic acid, or 1.5–25 mM tartaric acid) was added, and scanning continued for an additional 54 s approximately. The final concentrations and corresponding pH values of test solutions are shown in Table 1. The response was measured as the change in fluorescence intensity from the baseline to the peak value, expressed as delta relative fluorescence units (ΔRFU). For each 96-well plate, responses to 1 mM HCl were measured in four wells to calculate the mean ΔRFU (1 mM HCl). Each ΔRFU value was then divided by this mean to normalize the data, thereby reducing the potential variability caused by differences in transfection efficiency or other inter-plate factors.

**Table 1:** Concentrations and corresponding pH values of acids used in the membrane potential assay (Fig. 1).

Acid							
HCl	**mM**	0.3	1	3	5	10	
	pH	3.38	2.93	2.41	2.26	1.94	
Acetic acid	**mM**	0.3	1	3	5	10	
	pH	4.17	3.87	3.60	3.36	3.33	
Lactic acid	**mM**	0.3	1	3	5	10	30
	pH	3.84	3.41	3.13	3.03	2.80	2.49
Propionic acid	**mM**	0.3	1	3	5	10	
	pH	4.28	3.94	3.64	3.56	3.44	
Formic acid	**mM**	0.3	1	3	5	10	
	pH	3.87	3.45	3.16	2.99	2.70	
Butyric acid	**mM**	0.3	1	3	5		
	pH	4.18	3.92	3.52	3.43		
Tartaric acid	**mM**	0.3	1	3	5	10	
	pH	3.43	3.15	2.79	2.62	2.42	
Malic acid	**mM**	0.3	1	3	5	10	
	pH	3.59	3.28	2.92	2.76	2.62	
Citric acid	**mM**	0.1	0.3	1	3	5	
	pH	3.83	3.30	2.99	2.66	2.53	

EC_50_ values and their 95 % confidence intervals were calculated using IGOR Pro (version 6.34J, WaveMetrics, Portland, OR) (Table 2). EC_50_ values were determined by fitting individual normalized responses (ΔRFU/ΔRFU (1 mM HCl)) to the Hill equation using nonlinear least-squares regression. The standard error of each EC_50_ estimate was obtained from the parameter covariance matrix produced during curve fitting, and the 95 % confidence interval was calculated as EC_50_ ± 1.96 × standard error.

**Table 2:** Calculated EC_50_ values with 95 % confidence intervals (CI) in the membrane potential assay (Fig. 1).

Acid	EC_50_ (mM)	95 % CI (Lower)	95 % CI (Upper)
Citric acid	0.74	0.31	1.16
Tartaric acid	1.16	0.24	2.08
Malic acid	1.59	0.64	2.54
Formic acid	1.85	1.13	2.58
Butyric acid	1.89	0.72	3.06
Propionic acid	3.20	1.67	4.73
Acetic acid	3.73	−0.30	7.77
HCl	3.84	3.15	4.53
Lactic acid	7.57	2.53	12.60

Note: For acetic acid, the lower bound of the 95 % confidence interval crossed zero, reflecting variability in curve fitting.

### 2.4 Electrophysiology

Whole-cell patch clamp recordings were performed as in our previous reports [14,15]. Bovine OTOP1/pcDNA3.1(+) was co-transfected with EGFP/pCAGGS into HEK293T cells using ScreenFect A. We selected only EGFP-positive cells for electrophysiological recordings to ensure OTOP1 expression. In addition, EGFP-expressing cells that were mock-transfected with pcDNA3.1(+) and EGFP were used as negative controls. The standard and acidic bath solutions contained 140 mM NaCl, 5 mM KCl, 10 mM HEPES, 2 mM MgCl_2_, 2 mM CaCl_2_ and 10 mM glucose. The pH of acidic bath solutions (pH 3.0–5.5) was adjusted using HCl. Since the human ASIC1a channel in HEK293T cells can be blocked by a pH 6.8 bath solution [16], we adjusted the standard bath solution to pH 6.8 with NaOH. In Fig. 2C–F, we used citric acid or HCl solutions with or without 1–30 mM ZnCl_2_. The pH of these solutions was adjusted to pH 4.5 with citric acid or HCl. After addition of 30 mM ZnCl_2_ to the citric acid solution (nominal pH 4.5), the pH was measured and found to be 4.39, indicating only a minimal change in pH. Cells were voltage-clamped at −60 mV, and currents were recorded using an EPC10 amplifier (HEKA Elektronik, Lambrecht, Germany) controlled by PatchMaster software (HEKA Elektronik). Capacitive transients were compensated by adjusting the C-fast and C-slow parameters. Series resistance was compensated by 60 % with a time constant of 100 μs. Leak subtraction was not applied. Recorded data were analyzed using IGOR Pro. Data were sampled at 10 kHz and filtered at 2 kHz. Patch pipettes had resistances between 2.5 and 5 MΩ, and they were filled with a pipette solution consisting of 140 mM KCl, 5 mM EGTA, and 10 mM HEPES. We adjusted the pipette solution to pH 7.4 with KOH.

### 2.5 Statistical analysis

For the membrane potential assay, data were obtained from four wells per condition (technical replicates) within a single experiment. For patch-clamp recordings, data were obtained from individual cells recorded under the same experimental conditions.

Normalized fluorescence responses (ΔRFU/ΔRFU (1 mM HCl)) and current densities were treated as parametric data. Because the homogeneity of variance assumption was violated in several datasets, group differences among acids were evaluated using Welch's ANOVA, which is robust to unequal variances. Post hoc pairwise comparisons were performed using the Games–Howell test, which does not assume equal variances or equal sample sizes. These analyses were conducted using IBM SPSS Statistics (Version 27, Armonk, NY, USA). Statistical significance was set at *P* < 0.05.

## 3 Results

### 3.1 Acidic compounds dose-dependently activated bovine OTOP1 in membrane potential assay

**Fig. 1:**
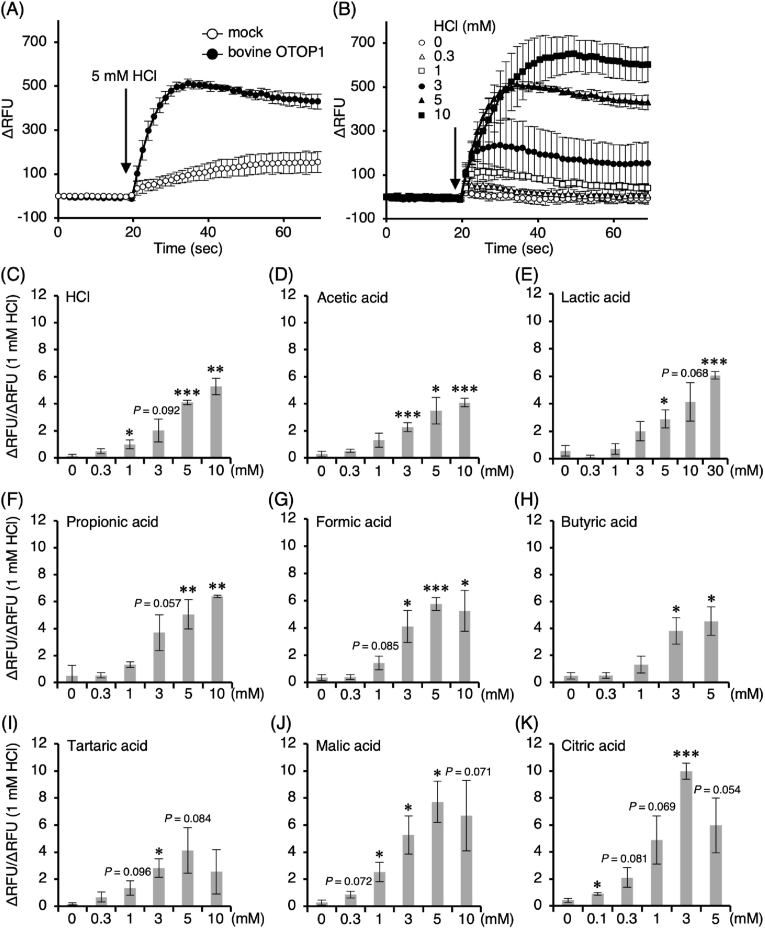
Acid-induced membrane depolarization in HEK293T cells expressing bovine OTOP1. (A) Changes in fluorescence intensity of a membrane potential–sensitive dye (ΔRFU) in mock-transfected cells and bovine OTOP1–expressing cells following application of 5 mM HCl. (B) Representative time courses of membrane potential–sensitive dye fluorescence in bovine OTOP1–expressing cells in response to increasing concentrations of HCl. (C) Peak normalized fluorescence responses (ΔRFU/ΔRFU induced by 1 mM HCl) corresponding to the responses shown in (B). (D–K) Concentration-dependent effects of various acids on normalized membrane potential–sensitive dye fluorescence in bovine OTOP1–expressing cells. Peak normalized responses after acid application are shown. Data are presented as mean ± SD from four wells per condition (n = 4 technical replicates). Statistical significance was evaluated using Welch's ANOVA followed by Games–Howell post hoc tests, with comparisons made against 0 mM for each acid. Asterisks indicate statistically significant differences (∗*P* < 0.05, ∗∗*P* < 0.01, ∗∗∗*P* < 0.001), whereas exact *P* values are shown directly in the figure for comparisons with 0.05 ≦ *P* < 0.1. Arrows indicate the timing of acid application.

First, we confirmed that the fluorescence intensity increased with 5 mM HCl administration in bovine OTOP1-expressing cells, while no increase was observed in mock cells (Fig. 1A). Secondly, in bovine OTOP1-expressing cells, HCl dose-dependently activated bovine OTOP1 (Fig. 1B). The ΔRFU/ΔRFU (1 mM HCl) values of these responses are shown in Fig. 1C. One to 10 mM HCl significantly increased the fluorescence values compared to 0 mM HCl (vehicle; consisting of 1 × HBSS buffer with water = 17:3). These results suggest that HCl dose-dependently activates bovine OTOP1.

Besides HCl, other acidic compounds (acetic, lactic, propionic, formic, butyric, tartaric, malic, and citric acids) also dose-dependently activated bovine OTOP1 (Fig. 1D–K). Based on these data, EC_50_ values with 95 % confidence intervals were calculated and are summarized in Table 2. These acidic compounds are found in the rumen and in the feed in cattle, suggesting that many acidic compounds acting on cattle taste buds activate OTOP1.

### 3.2 HCl activated bovine OTOP1 pH-dependently in whole-cell patch clamp test

**Fig. 2:**
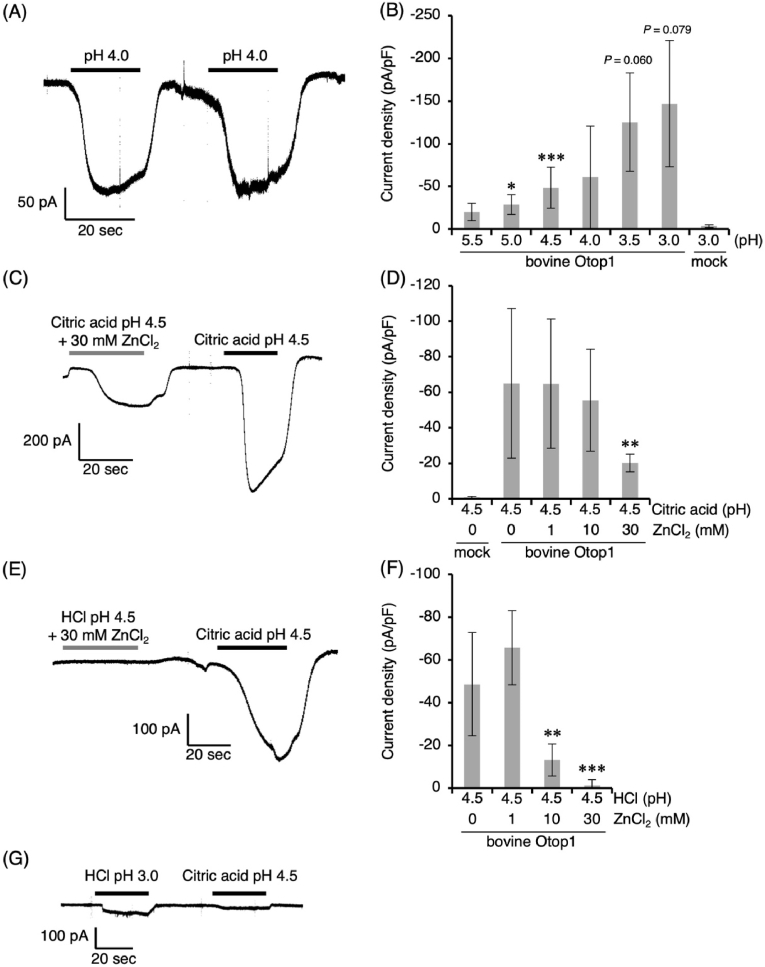
Whole-cell patch-clamp analysis of acid activation and zinc inhibition of bovine OTOP1. All recordings were performed at a holding potential of −60 mV. (A) Representative whole-cell currents recorded from bovine OTOP1–expressing HEK293T cells in response to HCl (pH 4.0). (B) Peak current densities evoked by HCl at different pH values. Mock cells were not activated by HCl (pH 3.0). (C) Representative whole-cell currents recorded in response to citric acid (pH 4.5) in the presence of 30 mM ZnCl_2_, followed by citric acid (pH 4.5) alone. (D) Concentration-dependent inhibition of citric acid (pH 4.5)–evoked currents by ZnCl_2_ in bovine OTOP1–expressing cells. Mock cells were not activated by citric acid (pH 4.5). (E) Representative whole-cell currents recorded in response to HCl (pH 4.5) in the presence of 30 mM ZnCl_2_, followed by citric acid (pH 4.5) alone. (F) Concentration-dependent inhibition of HCl (pH 4.5)–evoked currents by ZnCl_2_ in bovine OTOP1–expressing cells. (G) Representative whole-cell currents recorded from mock cells in response to HCl (pH 3.0), followed by citric acid (pH 4.5). Data are presented as mean ± SD from individual cells (n = 3–13 cells in B; n = 3–14 cells in D; n = 4–13 cells in F). Statistical significance was assessed using Welch's ANOVA followed by Games–Howell post hoc tests, with comparisons made against pH 5.5 in (B), citric acid (pH 4.5) without ZnCl_2_ in (D), and HCl (pH 4.5) without ZnCl_2_ in (F). Asterisks indicate statistically significant differences (∗*P* < 0.05, ∗∗*P* < 0.01, ∗∗∗*P* < 0.001), whereas exact *P* values are shown directly in the figure for comparisons with 0.05 ≦ *P* < 0.1.

Next, we examined whether acidic compounds activate the bovine OTOP1 channel using a whole-cell patch clamp test. The HCl solution (pH 4.0) activated the bovine OTOP1 channel and induced inward currents repeatedly (Fig. 2A). The current densities induced by HCl in bovine OTOP1-expressing cells increased in a pH-dependent manner (Fig. 2B). Since mock-transfected cells showed little or no response to acid stimulation (pH 3.0), the observed currents are attributable to bovine OTOP1 expression (Fig. 2B and G).

### 3.3 ZnCl_2_ inhibited bovine OTOP1

In the whole-cell patch clamp test, we confirmed that 30 mM ZnCl_2_ inhibited the bovine OTOP1 current induced by citric acid (pH 4.5, Fig. 2C). However, no inhibition was observed at concentrations between 1 and 10 mM (Fig. 2D). The inhibition of bovine OTOP1 by ZnCl_2_ was dose-dependent (Fig. 2D). We also confirmed that the mock-transfected cells did not respond to citric acid at pH 4.5 (Fig. 2D and G). In addition, ZnCl_2_ exhibited inhibitory effects on HCl at pH 4.5 starting at 10 mM, and completely suppressed the response at 30 mM (Fig. 2E and F). Zinc inhibited currents evoked by both citric acid and HCl, indicating that zinc acts, at least in part, on bovine OTOP1 itself rather than indirectly affecting solution chemistry.

## 4 Discussion

The present results suggest that bovine OTOP1 is a functional receptor for various sour taste substances, and they indicate that zinc inhibits bovine OTOP1. The results also imply that this protein, which is thought to be expressed in the taste buds (although this has not yet been reported in cattle), is activated by acidic substances in grass, silage, and concentrate feed, as well as by various acidic substances that return to the oral cavity from the rumen during rumination. Bovine OTOP1 (Accession No. NP_001193713.1) is 78.08 % homologous to mouse OTOP1 (Accession No. NP_766297.2), and these OTOP1s respond similarly to acidic substances.

Zinc is an essential nutrient, and its deficiency in cattle results in a variety of symptoms, including reduced growth rate and immune function [17]. Because zinc is present in grass and concentrate feed, cattle consume zinc on a daily basis. Zinc is important for maintaining skin and keratin and is also effective in preventing mastitis [18] and hoof disease [19]. Supplemental zinc for dairy cows is usually in inorganic form (zinc oxide or zinc sulfate), and there are new sources of zinc, such as zinc nanoparticles [19]. In this study, since we showed that zinc inhibits bovine OTOP1, it is plausible that zinc may suppress sour taste signaling in cattle. However, the ZnCl_2_ concentrations required to inhibit bovine OTOP1 in this study (10–30 mM) are substantially higher than zinc levels typically encountered under physiological feeding conditions, indicating that zinc at near-physiological concentrations (∼1 mM) is unlikely to suppress sour taste signaling in cattle. These findings suggest that zinc may modulate OTOP1 activity and sour taste perception when applied at pharmacological concentrations. It has been reported that cattle show weak preference or strong avoidance for sour taste [1]. Adding zinc at pharmacological concentrations to some feed ingredients, particularly those with a strong sour taste and low palatability, may reduce sour taste perception and improve palatability for cattle. However, the present study does not provide direct evidence linking OTOP1 inhibition to feed intake or feeding behavior, and this possibility remains to be tested experimentally in future feeding and preference studies.

Despite sharing moderate sequence homology between bovine and mouse OTOP1 (78.08 %), their functional properties appear to differ. Previous studies have shown that mouse OTOP1 is strongly inhibited by approximately 1 mM ZnCl_2_ [12], whereas bovine OTOP1 required substantially higher ZnCl_2_ concentrations (≧10 mM) for comparable inhibition in our experiments. This difference suggests a divergence in zinc sensitivity between species, although the precise molecular determinants remain to be elucidated.

The EC_50_ values obtained for nine acids revealed a clear structure–activity relationship in bovine OTOP1 (Table 2). Polycarboxylic acids such as citric, tartaric, and malic acids activated OTOP1 at sub-millimolar to low millimolar concentrations, indicating markedly higher potency than monocarboxylic acids. In contrast, the major rumen-derived volatile fatty acids (acetic, propionic, and butyric acids) showed moderate sensitivity, whereas lactic acid exhibited the weakest activation. Notably, HCl, a strong inorganic acid, showed only intermediate potency, further suggesting that OTOP1 is not a simple proton sensor but integrates physicochemical properties specific to organic acids. These findings imply that bovine OTOP1 is tuned to detect plant-derived organic acids with high sensitivity, while showing reduced responsiveness to rumen-derived volatile fatty acids, potentially reflecting adaptation to the ruminant feeding ecology.

If OTOP1 is expressed in bovine taste cells as it is in mice, the activation of oral OTOP1 could be a key factor in regulating rumination and salivation. Ruminants maintain ruminal pH at around 5.5–6.5, which is suitable for rumen microbial activity, by neutralizing acids through the secretion of large amounts of saliva containing high concentrations of bicarbonate and phosphate, which have buffering effects [13]. It would be interesting to determine whether bovine OTOP1 is involved in these ruminant-specific physiological effects, but there is currently no evidence to support this.

Although the present study characterized the functional properties of bovine OTOP1 using a heterologous expression system, its expression in bovine taste buds has not yet been directly demonstrated. Future studies employing immunohistochemistry or *in situ* hybridization using anti-OTOP1 antibodies or OTOP1-specific probes will be necessary to confirm the localization of OTOP1 in bovine taste tissues, as well as in the ruminal epithelium. In addition, heterologous systems such as HEK293T cells may lack taste cell–specific cofactors or regulatory mechanisms that modulate OTOP1 function. Therefore, electrophysiological recordings and calcium imaging analyses using native bovine taste cells will be required to validate acid-evoked responses under physiological conditions. Finally, behavioral studies examining cattle feeding responses to acidic compounds will be essential to determine whether the *in vitro* responses observed in this study translate into sour taste perception and feeding behavior *in vivo*.

Accumulating evidence from recent studies has expanded our understanding of the proton channel OTOP1 beyond its originally proposed role as a sour taste receptor. Genome editing–based epitope tagging approaches have demonstrated that OTOP1 is localized at the apical membrane of taste receptor cells and is not strictly restricted to canonical type III “sour” cells, suggesting broader cellular distribution and accessibility to luminal stimuli [20]. In addition, post-translational regulation has emerged as an important factor in OTOP1 function, as N-glycosylation was shown to be required for proper plasma membrane targeting and maximal proton current activity [21]. Furthermore, recent pharmacological studies have identified small-molecule positive allosteric modulators that enhance OTOP1 gating in a pH-dependent manner, highlighting additional regulatory dimensions of this proton channel [22]. Whether these regulatory features—subcellular localization, post-translational modification, and chemical modulation—are conserved in bovine OTOP1 remains unknown. Future studies will be required to determine if bovine OTOP1 shares similar molecular regulatory mechanisms and whether such properties influence acid-evoked responses under physiologically relevant conditions in cattle.

The analysis of bovine single chorda tympani nerve fibers revealed four major taste-responsive clusters: fibers most responsive to salty taste, sour taste, short-chain fatty acids, and certain bitter substances [10]. Since cattle are primarily grass eaters, it is easy to understand that they do not have the most responsive nerves for sweetness. It is also reasonable that the nerves best for salty taste are present because cattle prefer rock salt. Moreover, since the various plants that bovines eat contain sour substances such as ascorbic acid and many bitter substances such as tannins and polyphenols, it makes sense that they would have nerves best for sour and bitter tastes. It is also logical that there are nerves best for short-chain fatty acids, as these enter the oral cavity from the rumen during rumination. Because each cluster responds to several tastes, including sweet and/or umami tastes, and each taste activates fibers of several clusters, Hellekant et al. posited that the taste qualities perceived by humans and cattle are different [10]. On this point, Ginane et al. emphasized the need to improve our understanding of taste perception in herbivores and noted that, until we understand how these animals categorize tastes, we can analyze only their preferences toward stimuli representing the five human taste qualities [1]. Considering the composition of bovine chorda tympani nerve fibers, a large proportion respond most strongly to sour stimuli. Thus, the finding that bovine OTOP1 responds to various acidic substances is important for understanding bovine taste physiology.

This is the first study to report a functional sour taste receptor in cattle. Specifically, bovine OTOP1 was shown to respond to various sour taste substances to which cattle are exposed to daily in the oral cavity.

## Declaration of generative AI and AI-assisted technologies in the manuscript preparation process

During the preparation of this manuscript, the authors used ChatGPT (OpenAI) to assist with language editing and improving the clarity of the text. After using this tool, the authors reviewed and edited the content as necessary and take full responsibility for the content of the published article.

## CRediT authorship contribution statement

**Fuminori Kawabata:** Conceptualization, Funding acquisition, Investigation, Project administration, Writing – original draft, Writing – review & editing. **Yuko Kawabata:** Investigation, Methodology, Project administration, Writing – original draft, Writing – review & editing.

## Declaration of competing interest

The authors declare that they have no known competing financial interests or personal relationships that could have appeared to influence the work reported in this paper.

## Data Availability

Data will be made available on request.

## References

[1] Ginane C., Baumont R., Favreau-Peigné A. (2011). Perception and hedonic value of basic tastes in domestic ruminants. Physiol. Behav..

[2] Nei M., Niimura Y., Nozawa M. (2008). The evolution of animal chemosensory receptor gene repertoires: roles of chance and necessity. Nat. Rev. Genet..

[3] Davies R.O., Kare M.R., Cagan R.H. (1979). Distribution of taste buds on fungiform and circumvallate papillae of bovine tongue. Anat. Rec..

[4] Tabata S., Wada A., Kobayashi T., Nishimura S., Muguruma M., Iwamoto H. (2003). Bovine circumvallate taste buds: taste cell structure and immunoreactivity to α-gustducin. Anat. Rec..

[5] Tabata S., Wada-Takemura A., Nishimura S., Iwamoto H. (2006). Structure of bovine fungiform taste buds and their immunoreactivity for gustducin. J. Vet. Med. Sci..

[6] Wong G.T., Gannon K.S., Margolskee R.F. (1996). Transduction of bitter and sweet taste by gustducin. Nature.

[7] He W., Yasumatsu K., Varadarajan V., Yamada A., Lem J., Ninomiya Y., Margolskee R.F., Damak S. (2004). Umami taste responses are mediated by α-transducin and α-gustducin. J. Neurosci..

[8] Gerlach K., Daniel J.L.P., Jobim C.C., Nussio L.G. (2021). A data analysis on the effect of acetic acid on dry matter intake in dairy cattle. Anim. Feed Sci. Technol..

[9] Bell F.R., Kitchell R.L. (1966). Taste reception in the goat, sheep and calf. J. Physiol..

[10] Hellekant G., Roberts T., Elmer D., Cragin T., Danilova V. (2010). Responses of single chorda tympani taste fibers of the calf (Bos taurus). Chem. Senses.

[11] Tu Y.H., Cooper A.J., Teng B., Chang R.B., Artiga D.J., Turner H.N., Mulhall E.M., Ye W., Smith A.D., Liman E.R. (2018). An evolutionarily conserved gene family encodes proton-selective ion channels. Science.

[12] Teng B., Wilson C.E., Tu Y.H., Joshi N.R., Kinnamon S.C., Liman E.R. (2019). Cellular and neural responses to sour stimuli require the proton channel OTOP1. Curr. Biol..

[13] Paudyal S. (2021). Using rumination time to manage health and reproduction in dairy cattle: a review. Vet. Q..

[14] Liang R., Kawabata Y., Kawabata F., Nishimura S., Tabata S. (2019). Differences in the acidic sensitivity of transient receptor potential vanilloid 1 (TRPV1) between chickens and mice. Biochem. Biophys. Res. Commun..

[15] Kawabata Y., Takai S., Sanematsu K., Yoshida R., Kawabata F., Shigemura N. (2023). The antiarrhythmic drug flecainide enhances aversion to HCl in mice. eNeuro.

[16] Gunthorpe M., Smith G., Davis J., Randall A. (2001). Characterisation of a human acid-sensing ion channel (hASIC1a) endogenously expressed in HEK293 cells. Pflügers Archiv.

[17] Miller W.J. (1970). Zinc nutrition of cattle: a review. J. Dairy Sci..

[18] Libera K., Konieczny K., Witkowska K., Żurek K., Szumacher-Strabel M., Cieslak A., Smulski S. (2021). The association between selected dietary minerals and mastitis in dairy cows—A review. Animals.

[19] Langova L., Novotna I., Nemcova P., Machacek M., Havlicek Z., Zemanova M., Chrast V. (2020). Impact of nutrients on the hoof health in cattle. Animals.

[20] Kaplan J.P., Ye W., Kileen H., Liang Z., Tran A., Chi J., Yang C., Cohen P., Liman E.R. (2025). Epitope tagging with genome editing in mice reveals that the proton channel OTOP1 is apically localized and not restricted to type III “sour” taste receptor cells. J. Neurosci..

[21] Sasaki O., Yano-Nashimoto S., Yamaguchi S. (2024). A proton channel, Otopetrin 1 (OTOP1) is N-glycosylated at two asparagine residues in third extracellular loop. J. Cell. Physiol..

[22] Kong X., Sun J., Zhang H., Yin Y., Liang X., Chen Y., Luo G., Xia H., Wang Y., Liu Z., Tang C. (2025). Preferential allosteric modulation of Otop1 channels by small molecule compounds. Commun. Biol..

